# The Light-Fueled Stable Self-Rolling of a Liquid Crystal Elastomer-Based Wheel

**DOI:** 10.3390/polym17040436

**Published:** 2025-02-07

**Authors:** Jinze Zha, Kai Li, Junxiu Liu

**Affiliations:** 1 College of Civil Engineering, Anhui Jianzhu University, Hefei 230601, China; zhajinze@stu.ahjzu.edu.cn (J.Z.); kli@ahjzu.edu.cn (K.L.); 2 Anhui Province Key Laboratory of Intelligent Geotechnics and Disaster Prevention, Anhui Jianzhu University, Hefei 230601, China

**Keywords:** self-excited motion, LCE, photothermal energy conversion, self-rolling, autonomous drive systems

## Abstract

Self-excited systems rely on stable external stimuli to initiate and sustain oscillations via internal processes. However, these oscillations can compromise system stability and increase friction, limiting their practical applications. To overcome this issue, we propose the light-fueled stable self-rolling of a liquid crystal elastomer (LCE)-based wheel. A photothermal response model based on an LCE was used to analyze the temperature distribution within the LCE rods. The driving torque for self-rolling is generated by the contraction resulting from the LCE’s photothermal response, which displaces the wheel’s center of mass. We then derived the equilibrium equations and identified the critical conditions for achieving stable self-rolling motion. Through the interaction between the temperature field and driving torque, the wheel achieves continuous and stable self-rolling by absorbing thermal energy to counteract damping dissipation. Numerical simulations revealed that the stable self-rolling velocity is influenced by several key parameters, including heat flux, the contraction coefficient, gravitational acceleration, the initial damping torque, and the rolling damping coefficient. The proposed LCE-based wheel enhances system stability and significantly reduces frictional losses. These characteristics make it a promising candidate for applications in autonomous drive systems, micro-transportation devices, and photothermal energy conversion technologies.

## 1. Introduction

Self-excited oscillation is a phenomenon in which a system exhibits periodic motion under a constant external stimulus [1,2]. The oscillatory behavior of such systems is primarily determined by their intrinsic parameters, which makes them highly robust to external disturbances and variations in system parameters [3]. Moreover, self-excited systems do not require periodic external stimuli to maintain motion. This significantly reduces the need for complex motion control and control system design [4]. These advantages make self-excited systems particularly promising for applications in fields such as autonomous robotics [5], medical equipment [6], and energy harvesting [7].

Recently, there has been growing interest in self-excited systems that incorporate stimuli-responsive smart materials. These materials encompass ionic gels [8], hydrated gels [9,10,11], liquid crystal elastomers (LCEs) [12,13,14,15,16,17], dielectric elastic materials [18], and thermally sensitive polymers. These materials exhibit efficient energy conversion, rapid response times, and multifunctionality. Such systems exhibit a wide range of motion behaviors, including rolling [19,20,21,22,23], vibration [24,25,26,27], rotation [28,29,30,31], bending [32,33,34,35], chaotic motion [36], beating [37], spinning [38], jumping [39,40,41], twisting [42,43], wobbling [44], and oscillating [45,46,47]. These dynamic motions are driven by complex interactions between physical and chemical processes, such as large-scale deformations and chemical responses [48], self-occlusion effects [49,50], and photothermal gradients in surface tension [51,52].

LCEs, due to their unique blend of liquid crystal and elastomeric properties, are ideal candidates for self-excited systems [53,54,55,56,57]. When exposed to external stimuli like heat [58,59,60], light [61,62,63], and magnetic fields [64,65], LCEs undergo large, reversible deformations. Their photothermal responsiveness offers precise, contactless control and high energy efficiency, and they have remote activation ability. These properties make LCEs highly promising for advanced self-excited systems and smart material applications [66,67,68].

However, despite significant advancements in LCE-based self-excited systems, their inherent oscillatory behaviors can sometimes compromise structural stability and increase friction [24,33]. For instance, the self-rotating system in [69] relies on light-induced bending for motion, but it still faces challenges related to rigid track constraints and friction management. Similarly, the LCE rod system in [70] achieves continuous rotation in microgravity. Yet, it suffers from high friction due to non-uniform deformation and structural complexity. It also requires precise light control, which limits its adaptability in dynamic environments. In contrast, our proposed light-fueled self-rolling mechanism greatly improves upon these limitations by leveraging a photothermal response model. The non-uniform temperature distribution within the wheel causes the contraction of LCE rods. This displaces the wheel’s center of mass and generates driving rolling torque. This results in a stable self-rolling motion that is independent of external constraints such as precise light control or track geometry. By absorbing thermal energy from constant illumination, the system counteracts damping dissipation, ensuring continuous motion with minimal friction. Theoretical analyses show that factors such as heat flux, contraction coefficients, and damping coefficients affect the system’s rolling velocity. During the stable self-rolling state, the wheel maintains an almost constant shape, minimizing friction and preventing oscillations. The self-rolling wheel proposed in this paper features a simple design, exceptional stability, and low friction. It is expected to show great potential for applications in fields that require high accuracy and stability, such as soft robotics, micro-actuators, and energy harvesting devices.

The structure of this paper is as follows: Section 2 analyzes the wheel’s temperature field using the LCE photothermal response model and derives the driving torque expression. Section 3 presents the equilibrium equation and examines the critical conditions for stable self-rolling. Section 4 investigates the impact of key parameters on stable self-rolling velocity. Section 5 considers the potential applications of the LCE wheel-driven vehicle. Finally, Section 6 concludes the study.

## 2. Mechanism of Stable Self-Rolling

In this section, we first present a schematic diagram of the LCE-based wheel under constant light exposure. Using the photothermal response model of an LCE, we analyze the temperature distribution in the LCE rods and derive the mathematical formulation for the driving rolling torque.

### 2.1. A Model of an LCE-Based Wheel

**Figure 1:**
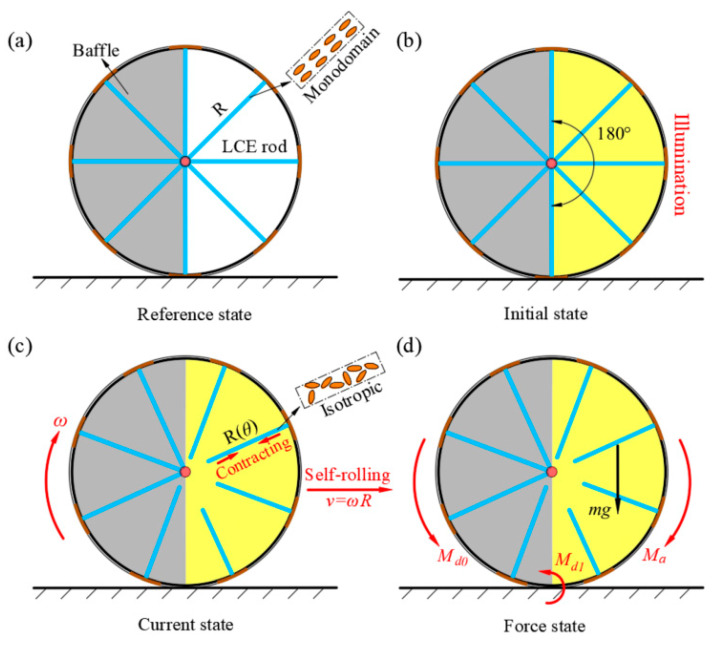
A schematic of the self-rolling LCE-based wheel under constant illumination. (**a**) The reference state. (**b**) The initial state. (**c**) The current state. (**d**) A force analysis. Under constant illumination, the non-uniform contraction of the LCE rods causes a shift in the wheel’s center of mass, thereby driving the wheel to roll forward.

Figure 1 shows a schematic diagram of the photothermally responsive LCE-based wheel under constant illumination. As depicted in Figure 1a, several LCE rods are fixed at one end to the wheel’s edge. The other end remains free, with an initial length of R. A baffle is placed on the left side of the wheel to block the illumination. The baffle’s center is fixed directly below the wheel and does not move with self-rolling motion. In the reference state, the azobenzene-based liquid crystalline mesogens within the LCE rods are aligned along the rod’s axis in a monodomain state. Assuming that the modulus of the LCE rods is sufficiently large, they are only allowed to contract axially without experiencing longitudinal bending. As shown in Figure 1b, while the entire wheel is exposed to illumination, the baffle blocks the illumination on the left side. Consequently, only the right half of the wheel (highlighted in yellow) is exposed to illumination. The illuminated region is denoted as $\theta =\left[0,\pi \right]$.

As shown in Figure 1c, when the LCE rods are exposed to the illuminated region, the photothermal effect causes the LCE rods to absorb thermal energy. This absorption induces a phase transition of the liquid crystalline mesogens from the monodomain state to an isotropic state, leading to axial contraction in the rods. At this point, the rod length becomes $R\left(\theta \right)$. Upon leaving the illuminated region, the mesogens recover to the monodomain state, reversing the contraction, and the rod length returns to R [71,72,73]. This photothermal response generates uneven strain across the LCE rods, resulting in a shift in the wheel’s center of mass. Under the influence of gravity, this shift in the center of mass produces a driving rolling torque $M_{a}$, as shown in Figure 1d. As the driving rolling torque $M_{a}$ increases, the rotational angular velocity of the wheel rises, resulting in a more uniform temperature distribution across the LCE rods. However, the increased angular velocity leads to a reduction in the contraction of the rods, which in turn decreases the $M_{a}$. This dynamic interaction between angular velocity, contraction, and torque is essential for sustaining self-rolling motion. The energy absorbed by the LCE rods through photothermal effects plays a key role in compensating for the energy lost due to damping forces. As the wheel rolls, damping forces lead to energy loss, which would normally slow down the motion. However, the thermal energy absorbed from the constant illumination is continuously converted into mechanical work through the photothermal response of the LCE rods. This process compensates for the energy lost to damping, thus maintaining the wheel’s motion.

### 2.2. Temperature Field Distribution of LCE Rods

As shown in Figure 1c, when the LCE rods enter the illuminated region, we assume that the temperature field within the rods remains nearly uniform at each angular position. This assumption holds because the cross-sectional radius of the LCE rods is much smaller than the light penetration depth. During stable self-rolling, heat exchange occurs between the LCE rods and the surrounding environment, resulting in a relatively low Biot number [74]. The temperature distribution within the LCE rods can be expressed by the following equation [75]:

(1) $$\frac{\partial T\left(\theta ,t\right)}{\partial t}+\vec{V}\left(\theta ,t\right)\nabla T\left(\theta ,t\right)=\frac{I\left(\theta \right)-KT\left(\theta ,t\right)}{\rho_{c}}$$

where $\rho_{c}$ and K represent the specific heat capacity and thermal conductivity coefficient. $\vec{V}\left(\theta ,t\right)$ denotes the velocity field of the LCE rod at a given time t, which can be expressed in terms of the rotational angular velocity $\vec{V}\left(\theta ,t\right)=\omega R\vec{e_{0}}$ of the LCE-based wheel. Additionally, $I\left(\theta \right)$ represents the heat flux at any angular position of the LCE rod.

Solving Equation (1) analytically is complex, so we introduce an activation function related to the angular position I(θ) to assist in the solution.

(2) $$I\left(\theta \right)=I_{0}\mathrm{sigmoid}\left[\alpha \theta \left(\theta -\pi \right)\right]$$

where $I_{0}$ represents the heat flux generated under illumination, and α describes the transition coefficient between the illuminated and non-illuminated regions. We define α=200 such that $I(\theta )=I_{0}$ within the illuminated region and I(θ)=0 outside of the illuminated region. The parameter α governs the smooth transition of I(θ) across the boundary between the illuminated and non-illuminated regions. Based on this, the following expression is derived:

(3) $$\left\{\begin{array}{c} I(\theta )=I_{0},0<\theta \leq \pi \\ I(\theta )=0,\pi <\theta \leq 2\pi \end{array}\right.$$

Under stable self-rolling conditions, the LCE-based wheel satisfies $T\left(\theta ,t\right)=T\left(\theta \right)$. Consequently, Equation (1) can be reformulated as follows:

(4) $$\omega \frac{dT(\theta )}{d\theta }=\frac{I_{0}\mathrm{sigmoid}[\alpha \theta (\theta -\pi )]-KT(\theta )}{\rho_{c}}$$

where the angular velocity ω can be expressed in terms of the self-rolling velocity v=ωR. By defining the dimensionless parameters as $\bar{\omega }=\omega \tau_{0}$, $\bar{v}=v\tau_{0}/R$, $\bar{I_{0}}=I_{0}/KT_{e}$, $\bar{T}=T/T_{e}$, and $\tau_{0}=\rho_{c}/K$ (where $\tau_{0}$ denotes the characteristic heat exchange time between the LCE rods and the environment), Equations (2) and (4) can be reformulated as follows:

(5) $$\bar{I}\left(\theta \right)=\bar{I_{0}}\mathrm{sigmoid}\left\{\alpha \theta \left[\theta -\pi \right]\right\}$$

(6) $$\bar{v}\frac{d\bar{T}\left(\theta \right)}{d\theta }=\bar{I_{0}}\mathrm{sigmoid}\left\{\alpha \theta \left[\theta -\pi \right]\right\}-\bar{T}\left(\theta \right)$$

By solving the governing differential equations, we obtain the following:

(7) $$\bar{T}\left(\theta \right)=\exp\left(-\frac{\theta }{\bar{v}}\right)c_{1}+\exp\left(-\frac{\theta }{\bar{v}}\right)\int_{0}^{\theta }\frac{\exp\left(\frac{\delta }{\bar{v}}\right)\left\{\bar{I_{0}}\mathrm{sigmoid}\left[\alpha \delta \left(\delta -\pi \right)\right]\right\}}{\bar{v}}d\delta$$

The constant $c_{1}$ is determined by the periodic boundary conditions, which ensures the continuity of the temperature field across the LCE rods:

(8) $$\bar{T}\left(0\right)=\bar{T}\left(2\pi \right)$$

**Figure 2:**
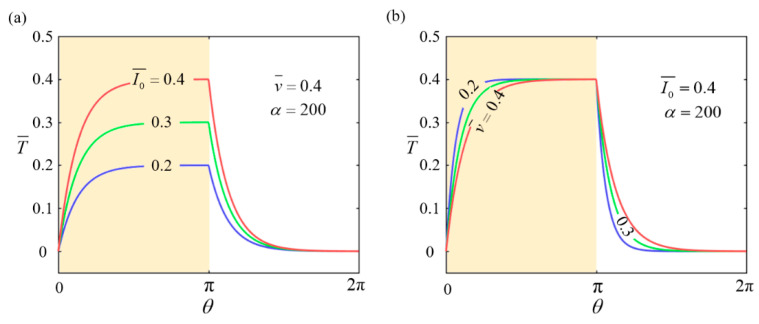
The temperature distribution $\bar{T}(\theta )$ in the LCE-based wheel. (**a**) The influence of the heat flux $\bar{I_{0}}$ on $\bar{T}(\theta )$. (**b**) The influence of the self-rolling velocity $\bar{v}$ on $\bar{T}(\theta )$. The temperature $\bar{T}(\theta )$ increases significantly with the increase in $\bar{I_{0}}$ or the decrease in $\bar{v}$.

Based on Equations (7) and (8), we can determine the temperature distribution within the LCE rods in the wheel. Figure 2 illustrates the effect of different parameter combinations on $\bar{T}(\theta )$ under stable self-rolling conditions. The yellow region in Figure 2 represents the region exposed to illumination. As depicted in Figure 2a, the temperature $\bar{T}(\theta )$ significantly increases as the heat flux $\bar{I_{0}}$ rises. This indicates that a higher $\bar{I_{0}}$ allows the LCE rods to absorb more thermal energy, resulting in a considerable increase in temperature. Specifically, as the heat flux increases, the photothermal effect of the LCE rods becomes more pronounced. This leads to higher local temperature peaks within the illuminated region. In Figure 2b, as the self-rolling velocity $\bar{v}$ decreases, the temperature $\bar{T}(\theta )$ rises progressively but eventually stabilizes at a peak value. At a lower $\bar{v}$, the LCE rods remain in the illuminated region for a longer duration, which allows them to absorb more heat and consequently increases the temperature. However, when $\bar{v}$ further decreases beyond a certain threshold, the extended exposure time in the illuminated region allows the absorbed thermal energy and dissipated heat to reach a dynamic equilibrium. This results in the temperature of the LCE rods to level off at the maximum value.

The effect of the transition coefficient α, which governs the gradient between the illuminated and non-illuminated regions, on the temperature distribution is negligible. This is because the primary factors influencing the temperature distribution are the heat flux $\bar{I_{0}}$ and the self-rolling velocity $\bar{v}$. The transition coefficient α merely determines the smoothness of the boundary gradient and does not significantly alter the overall temperature profile. As a result, the impact of α is not further explored in this study. For the sake of consistency, the conversion coefficient α is set to a constant value of 200 in the following analysis.

### 2.3. Gravitationally Induced Driving Rolling Torque

As shown in Figure 1c, each LCE rod undergoes a phase transition upon entering the illuminated region due to the effect of constant illumination exposure. This transition causes axial contraction of varying degrees in the liquid crystal medium depending on the angular position. When the length of the LCE rods shortens, the width simultaneously increases, ensuring that the overall volume and mass of the wheel remain constant. As the angle changes, non-uniform contraction leads to a shift in the wheel’s center of mass, generating a driving rolling torque under the influence of gravity. The resulting driving rolling torque can be described as

(9) $$M_{a}=\frac{mg}{4\pi }\int_{0}^{2\pi }[R\varepsilon (\theta )]\sin\theta d\theta$$

where m represents the total mass of all LCE rods, g is the gravitational acceleration, and ε(θ) denotes the photothermal response strain of the LCE rods, expressed as ε(θ)=[R−R(θ)]/R [76].

From the previous discussion, it is clear that the temperature of the LCE rods varies under different conditions, resulting in distinct contraction levels for each rod. For simplicity and to facilitate analysis, we assume a linear relationship between the photothermal response strain of the LCE rods and their temperature:

(10) $$\varepsilon \left(\theta \right)=C_{0}T\left(\theta \right)$$

where $C_{0}$ represents the contraction coefficient of the LCE rods. We assume the phase transition time is much smaller than the characteristic heat exchange time. This assumption is reflected by the dimensionless parameter g. This approach enables focusing on the steady-state behavior and simplifies the analysis. Similarly, we define the following dimensionless parameters: $\bar{M_{a}}=M_{a}{\tau_{0}}^{2}/mR^{2}$, $\bar{g}=g{\tau_{0}}^{2}/R$, $\bar{C_{0}}=C_{0}T_{e}$. Equation (9) can therefore be rewritten in its dimensionless form:

(11) $$\bar{M_{a}}=\frac{\bar{g}}{4\pi }\int_{0}^{2\pi }\bar{C_{0}}\bar{T}\left(\theta \right)\sin\theta d\theta$$

Using Equations (7) and (8) to calculate $\bar{T}\left(\theta \right)$, we proceed to investigate the effects of $\bar{I_{0}}$, $\bar{C_{0}}$, and $\bar{g}$ on the driving rolling torque $\bar{M_{a}}$.

**Figure 3:**
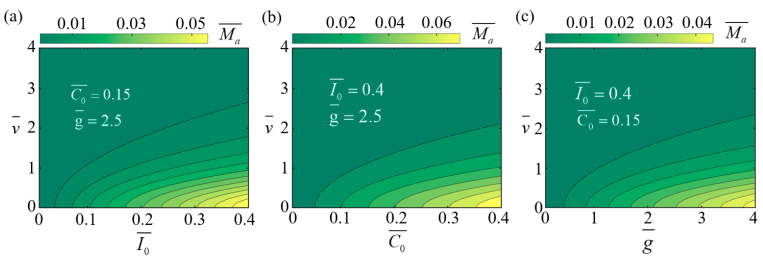
The influence of parameters $\bar{I_{0}}$, $\bar{C_{0}}$, and $\bar{g}$ on the driving rolling torque $\bar{M_{a}}$. (**a**) A contour plot of $\bar{M_{a}}$ with changes in $\bar{v}$ and $\bar{I_{0}}$. (**b**) A contour plot of $\bar{M_{a}}$ with changes in $\bar{v}$ and $\bar{C_{0}}$. (**c**) A contour plot of $\bar{M_{a}}$ with changes in $\bar{v}$ and $\bar{g}$. The driving torque $\bar{M_{a}}$ increases with increasing $\bar{I_{0}}$, $\bar{C_{0}}$, and $\bar{g}$ values and decreases with an increasing $\bar{v}$ value.

Figure 3 illustrates the changes in the driving rolling torque $\bar{M_{a}}$ under different parameter conditions. The specific parameter settings are provided in the figure. Figure 3a–c show the trends of $\bar{M_{a}}$ as variations in $\bar{I_{0}}$, $\bar{C_{0}}$, and $\bar{g}$, respectively. The parameters $\bar{I_{0}}$ and $\bar{C_{0}}$ describe the efficiency of thermal energy absorption by the LCE rods, while $\bar{g}$ reflects the effect of gravity on the wheel’s driving process. As observed, the driving rolling torque $\bar{M_{a}}$ increases with increasing $\bar{I_{0}}$, $\bar{C_{0}}$, and $\bar{g}$ values. This indicates that higher thermal energy input efficiency and a stronger gravitational effect enhance the driving torque. Moreover, the results also show that as the wheel’s self-rolling velocity $\bar{v}$ increases, the driving torque $\bar{M_{a}}$ decreases significantly. This is because, at a higher self-rolling velocity, the LCE rods spend a shorter duration in the illuminated region, thereby limiting heat absorption and reducing the effectiveness of the driving torque.

## 3. Critical Condition for Triggering Stable Self-Rolling

Based on the previous discussions, we derived and calculated the driving rolling torque $\bar{M_{a}}$, which is significantly influenced by parameters $\bar{I_{0}}$, $\bar{C_{0}}$, and $\bar{g}$. When the LCE-based wheel attains a stable self-rolling state, the angular acceleration drops to zero, and the shape of the LCE rods remains constant at any given moment, maintaining a periodic and steady condition. Under this condition, the motion of the wheel is governed by the balance between the driving rolling torque $\bar{M_{a}}$ and the total damping torque $\bar{M_{d}}$, as displayed in Figure 1d.

### 3.1. Equilibrium Equations

**Figure 4:**
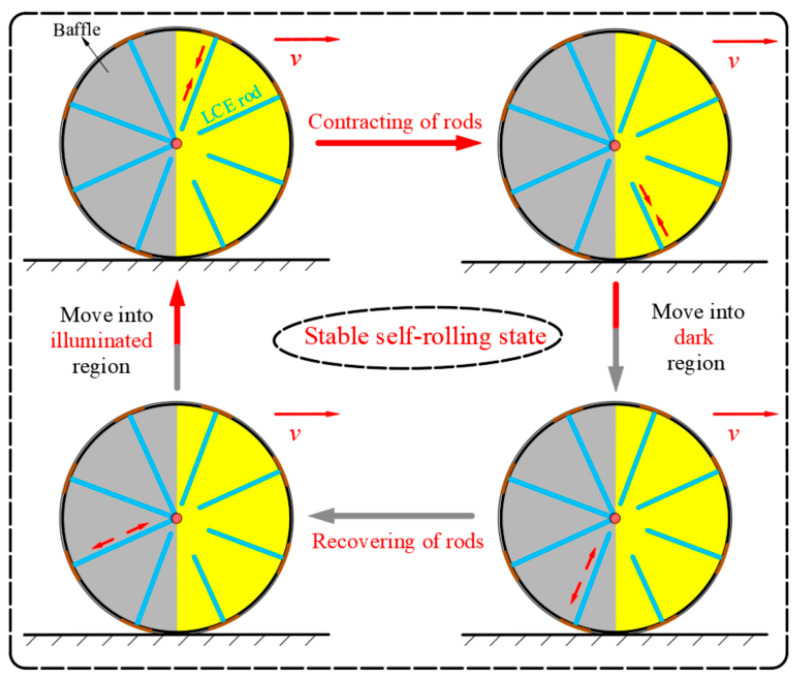
The stable self-rolling process of the LCE-based wheel. Under constant illumination, the LCE rods undergo periodic changes due to photothermal response-induced strain.

Figure 4 illustrates the stable self-rolling process of the LCE-based wheel within one complete cycle. The yellow region indicates the illuminated region. As the LCE rods move into the illuminated region, they gradually contract due to the photothermal effect. Upon moving into the dark region, the rods gradually return to their initial lengths, resulting in a shift in the center of mass. This shift generates the driving rolling torque that propels the wheel forward. An increase in self-rolling velocity enhances the uniformity of the temperature distribution across the LCE rods. This leads to nearly consistent contraction in each rod, minimizing internal inconsistencies within the system. Through the synergistic interaction of the driving torque and the thermal field, the wheel adjusts itself to an equilibrium state, maintaining a stable self-rolling velocity.

The torque balance equation for the LCE-based wheel is as follows:

(12) $$M_{a}=M_{d}$$

where the total damping torque consists of the initial damping torque $M_{d0}$ and rolling damping torque $M_{d1}$. $M_{d0}$ is a constant independent of the self-rolling motion. $M_{d1}$ is expressed as $M_{d1}=mg\beta$, where β is the rolling damping coefficient.

By nondimensionalizing the equation and introducing key dimensionless parameters $\bar{M_{d0}}=M_{d0}{\tau_{0}}^{2}/mR^{2}$ and $\bar{\beta }=\beta /R$, the torque balance equation is simplified to the following form:

(13) $$\bar{M_{a}}=\bar{M_{d0}}+\bar{g}\bar{\beta }$$

To study the self-rolling behavior of the LCE-based wheel under constant illumination, it is crucial to determine the typical values of the dimensionless parameters. Based on previous experimental results [20,77,78], Table 1 provides a summary of the material properties and geometric parameters of typical LCE rods, while Table 2 presents the corresponding dimensionless parameters derived from these properties. These values provide the theoretical foundation for analyzing the photothermal response characteristics and self-rolling behavior of the LCE-based wheel.

To solve the above equations using MATLAB, parameters $\bar{I_{0}}$ and $\bar{v}$ are first substituted into Equations (7) and (8) to calculate the temperature distribution $\bar{T}(\theta )$. Then, the resulting $\bar{T}(\theta )$ values are substituted into Equation (11) to compute the driving rolling torque $\bar{M_{a}}$. When the condition in Equation (13) is satisfied, meaning the driving rolling torque balances the damping torque, the LCE-based wheel is considered to have achieved a stable self-rolling state.

### 3.2. Critical Condition for Stable Self-Rolling

This section outlines the method for determining the critical driving torque ${\bar{M}}_{a}^{\lim}$ necessary for stable self-rolling. As discussed earlier, when the LCE-based wheel reaches a stable self-rolling state, the critical torque ${\bar{M}}_{a}^{\lim}$ needed to sustain this motion can be obtained. By combining Equation (4) with the variations in heat flux distribution between the illuminated and non-illuminated regions, and incorporating Equations (9) and (11), the specific expression for the critical driving torque ${\bar{M}}_{a}^{\lim}$ is derived as follows:

(14) $${\bar{M}}_{a}^{\lim}=\frac{\bar{g}\bar{C_{0}}\bar{I_{0}}}{2\pi }$$

**Figure 5:**
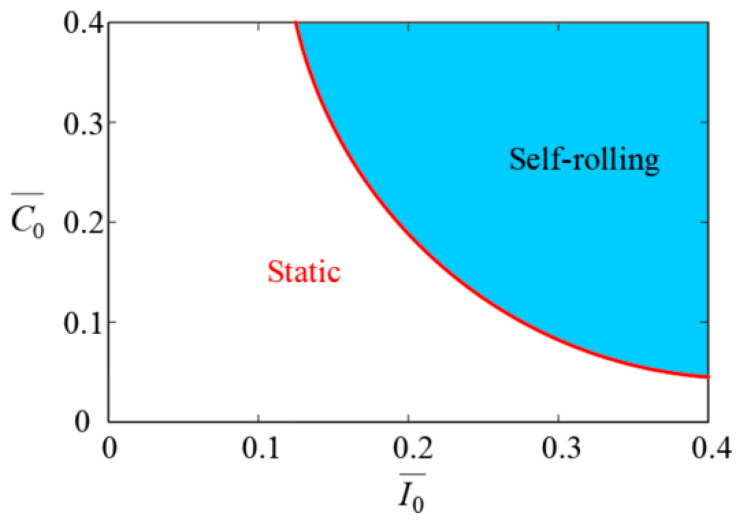
Phase diagram of $\bar{C_{0}}$ and $\bar{I_{0}}$ based on Equation (14), with other parameters set as $\bar{g}=2.5$, $\bar{M_{d0}}=0.005$, and $\bar{\beta }=0.001$. Wheel achieves stable self-rolling when combination of $\bar{C_{0}}$ and $\bar{I_{0}}$ lies in blue region; otherwise, wheel remains static.

Through Equation (14), the critical condition for the LCE-based wheel to achieve stable self-rolling can be calculated. An analysis reveals that this critical condition depends on the parameters set as $\bar{I_{0}}$, $\bar{C_{0}}$, and $\bar{g}$. By defining other parameters $\bar{g}=2.5$, $\bar{M_{d0}}=0.005$, and $\bar{\beta }=0.001$, the phase diagram of $\bar{C_{0}}$ and $\bar{I_{0}}$ is plotted, as shown in Figure 5. When the combination of $\bar{C_{0}}$ and $\bar{I_{0}}$ lies within the blue area, the photothermally induced driving torque is sufficient to overcome the damping torque. This enables the wheel to maintain a stable self-rolling state. Conversely, if the combination lies within the white region, the driving torque is insufficient to sustain self-rolling, and the wheel remains static.

## 4. Velocity of Stable Self-Rolling

In the preceding discussion, we derived the equilibrium equation and the critical conditions for stable self-rolling. In this section, we further combine the equilibrium equation with the critical conditions to explore the influence of key parameters on stable self-rolling behavior. We analyze factors such as heat flux, the contraction coefficient, gravitational acceleration, and damping characteristics. By doing so, we uncover how these parameters interact to affect the stable self-rolling state of the LCE-based wheel. This analysis provides a systematic theoretical foundation for understanding the self-rolling mechanism. Additionally, it offers valuable insights for optimizing design and parameter configuration in practical applications.

### 4.1. Effect of Heat Flux

**Figure 6:**
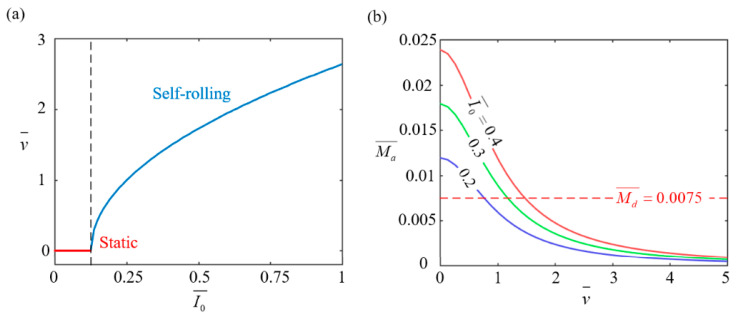
Effect of heat flux $\bar{I_{0}}$ on self-rolling characteristics of LCE-based wheel. (**a**) Relationship between self-rolling velocity $\bar{v}$ and heat flux $\bar{I_{0}}$. (**b**) Changes in driving rolling torque $\bar{M_{a}}$ with $\bar{v}$ for $\bar{I_{0}}$ values of 0.4, 0.3, and 0.2. When $\bar{I_{0}}$ exceeds 0.1255, self-rolling velocity increases with increasing $\bar{I_{0}}$.

Figure 6 illustrates the effect of heat flux $\bar{I_{0}}$ on the stable self-rolling behavior of the LCE-based wheel with parameters set to $\bar{C_{0}}=0.15$, $\bar{g}=2.5$, $\bar{M_{d0}}=0.005$, and $\bar{\beta }=0.001$. As depicted in Figure 6a, the self-rolling velocity of the wheel increases as the heat flux increases. An increase in heat flux causes the LCE rods to absorb more thermal energy, leading to a stronger contraction effect. This directly increases the driving torque, which accelerates self-rolling motion and ultimately raises the self-rolling velocity. When the heat flux is below 0.1255, the photothermal-induced driving torque is too weak to overcome the damping torque, causing the wheel to stay static. This threshold matches the theoretical value obtained from Equation (14). Figure 6b shows the relationship between self-rolling velocity and heat flux for different values (0.4, 0.3, and 0.2). As the heat flux increases, the driving torque decreases because higher speeds reduce the time the LCE rods spend in the illuminated region, limiting thermal energy absorption. The dashed line represents the damping torque, and its intersection with the solid line indicates the equilibrium point, marking the onset of stable self-rolling. Optimizing heat flux is essential for maintaining this balance, ensuring both efficient energy absorption and stable motion. In practical applications, fine-tuning the heat flux is crucial for achieving optimal performance, particularly in systems requiring precise control over motion stability and energy efficiency.

### 4.2. Effect of Contraction Coefficient

**Figure 7:**
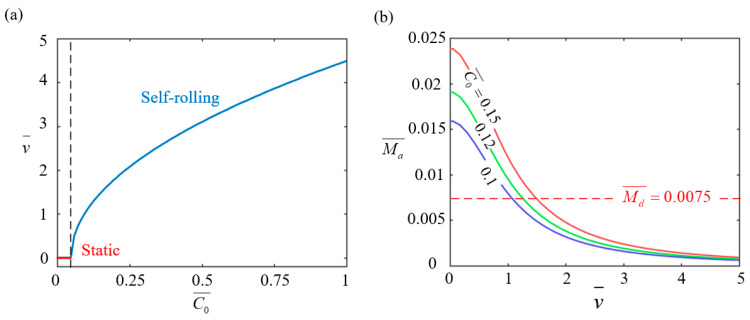
The effect of the contraction coefficient $\bar{C_{0}}$ on the self-rolling characteristics of the LCE-based wheel. (**a**) The relationship between the self-rolling velocity $\bar{v}$ and contraction coefficient $\bar{C_{0}}$. (**b**) Changes in the driving rolling torque $\bar{M_{a}}$ with $\bar{v}$ for $\bar{C_{0}}$ values of 0.15, 0.12, and 0.1. When $\bar{C_{0}}$ exceeds 0.0469, the self-rolling velocity increases with an increasing $\bar{C_{0}}$ value.

Figure 7 illustrates the effect of the contraction coefficient $\bar{C_{0}}$ on the stable rolling behavior of the LCE-based wheel with parameters set as $\bar{I_{0}}=0.4$, $\bar{g}=2.5$, $\bar{M_{d0}}=0.005$, and $\bar{\beta }=0.001$. As depicted in Figure 7a, the self-rolling velocity increases with the contraction coefficient. An increase in the contraction coefficient intensifies the photothermal contraction effect of the LCE rods, causing greater deformation of the material upon heating. This increased deformation directly enhances the driving torque, which accelerates self-rolling motion and ultimately leads to an increase in the self-rolling velocity. When the coefficient is below 0.0469, the driving torque is not enough to overcome the damping torque, causing the wheel to stay stationary. This critical value matches the theoretical prediction obtained from Equation (14). Figure 7b shows the relationship between the driving torque and self-rolling velocity for different contraction coefficients (0.15, 0.12, and 0.1). As the coefficient increases, the driving torque decreases because the LCE rods spend less time in the illuminated region, limiting thermal energy absorption and weakening the contraction effect. The dashed lines represent the damping torque, and the intersections with the driving torque curves mark the equilibrium points for stable self-rolling. In practical applications, optimizing the contraction coefficient is essential to balance the driving torque and damping forces. A higher contraction coefficient enhances the photothermal contraction effect, increasing the driving torque and self-rolling velocity, which leads to more stable and efficient motion.

### 4.3. Effect of Gravitational Acceleration

**Figure 8:**
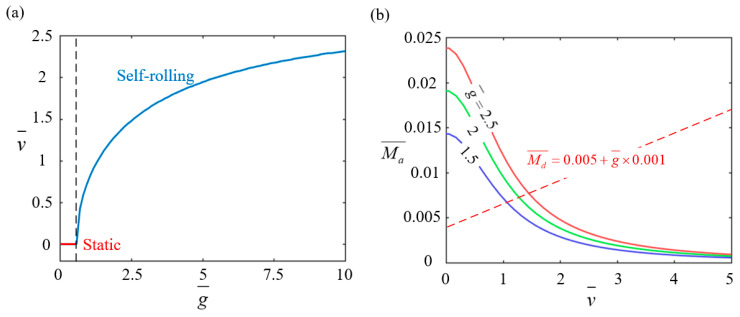
The effect of gravitational acceleration $\bar{g}$ on the self-rolling characteristics of the LCE-based rod wheel. (**a**) The relationship between the self-rolling velocity $\bar{v}$ and gravitational acceleration $\bar{g}$. (**b**) Changes in the driving rolling torque $\bar{M_{a}}$ with $\bar{v}$ for $\bar{g}$ values of 2.5, 2, and 1.5. When $\bar{g}$ exceeds 0.5847, the self-rolling velocity increases with an increasing $\bar{g}$ value.

Figure 8 illustrates the effect of gravitational acceleration $\bar{g}$ on the rolling behavior of the LCE-based wheel with parameters set as $\bar{I_{0}}=0.4$, $\bar{C_{0}}=0.15$, $\bar{M_{d0}}=0.005$, and $\bar{\beta }=0.001$. As depicted in Figure 8a, the self-rolling velocity increases with gravitational acceleration. An increase in gravitational acceleration results in a stronger gravitational force acting on the wheel, causing a shift in the wheel’s center of mass. This shift generates a larger driving force, which accelerates the wheel’s rolling motion and ultimately increases the self-rolling velocity. When the acceleration is below 0.5847, the driving torque from gravity is not enough to overcome the damping torque, keeping the wheel static. This critical value matches the theoretical prediction from Equation (14). Figure 8b shows the relationship between the driving torque and self-rolling velocity for different gravitational accelerations (2.5, 2, and 1.5). As acceleration increases, the driving torque decreases because the LCE rods spend less time in the illuminated region, reducing thermal energy absorption and weakening the contraction effect. The dashed lines represent the damping torque, $\bar{M_{d}}=0.005+g\times 0.001$, which increases with acceleration, indicating that the rolling damping torque is proportional to gravitational acceleration. The intersections of the driving torque curves with the damping torque lines mark equilibrium points for stable self-rolling. In practical applications, optimizing gravitational acceleration is crucial for balancing driving torque and damping torque to maintain stable self-rolling motion. While increasing gravitational acceleration enhances the driving torque and self-rolling velocity, the corresponding rise in damping torque must be carefully considered to avoid instability.

### 4.4. Effect of Initial Damping Torque

**Figure 9:**
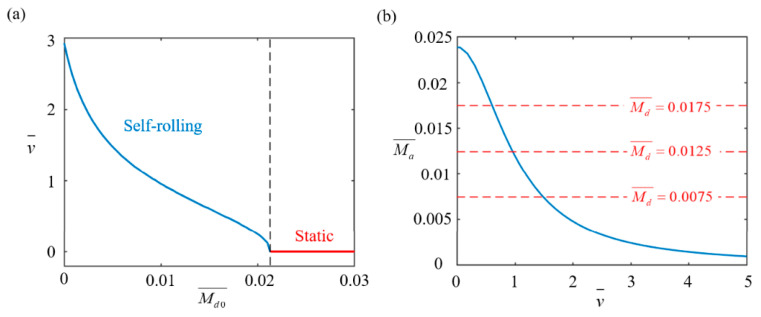
Effect of initial damping torque $\bar{M_{d0}}$ on self-rolling characteristics of LCE-based rod wheel. (**a**) Relationship between self-rolling velocity $\bar{v}$ and initial damping torque $\bar{M_{d0}}$. (**b**) Changes in driving rolling torque $\bar{M_{a}}$ with $\bar{v}$ for $\bar{M_{d0}}$ values of 0.005, 0.0075 and 0.01. When $\bar{M_{d0}}$ exceeds 0.0213, self-rolling velocity decreases with increasing $\bar{M_{d0}}$.

Figure 9 illustrates the effect of the initial damping torque $\bar{M_{d0}}$ on the self-rolling behavior of the LCE-based wheel with parameters set as $\bar{I_{0}}=0.4$, $\bar{C_{0}}=0.15$, $\bar{g}=2.5$, and $\bar{\beta }=0.0025$. As depicted in Figure 9a, the self-rolling velocity decreases with an increasing initial damping torque. When the other initial conditions remain unchanged, an increase in the initial damping torque generates additional resistance to the wheel’s motion. The increased damping torque leads to greater energy dissipation, which suppresses the acceleration of the wheel, ultimately resulting in a decrease in the self-rolling velocity. When it exceeds 0.0213, the driving torque is not enough to overcome the damping torque, and the wheel remains static. This critical value matches the theoretical prediction obtained from Equation (14). Figure 9b shows the relationship between the driving torque and self-rolling velocity for different initial damping torques (0.005, 0.0075, and 0.01). As the initial damping torque increases, the self-rolling velocity decreases due to reduced thermal energy absorption. The red dashed lines represent the total damping torque, consisting of the initial and rolling damping torques, with values of 0.0075, 0.0125, and 0.0175. The intersections of the driving torque curves with the red dashed lines show equilibrium points, where the driving torque balances the total damping torque for stable self-rolling. In practical applications, controlling the initial damping torque is critical for optimizing self-rolling behavior. A larger initial damping torque reduces the self-rolling velocity and may even prevent it. Therefore, careful control of this parameter is crucial for maintaining stable motion and reliability.

### 4.5. Effect of Rolling Damping Coefficient

**Figure 10:**
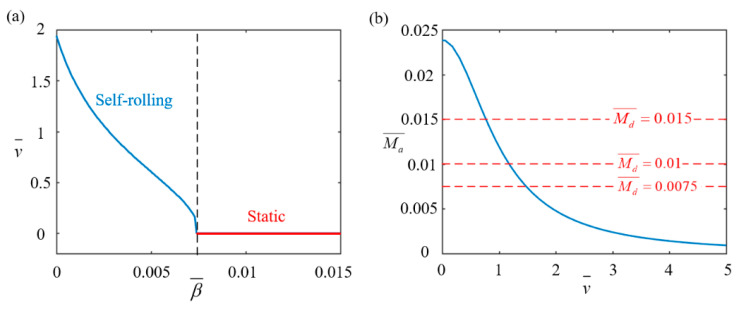
Effect of self-rolling damping coefficient $\bar{\beta }$ on self-rolling characteristics of LCE-based wheel. (**a**) Relationship between self-rolling velocity $\bar{v}$ and rolling damping coefficient $\bar{\beta }$. (**b**) Changes in driving rolling torque $\bar{M_{a}}$ with $\bar{v}$ for $\bar{\beta }$ values of 0.001, 0.002 and 0.004. When $\bar{\beta }$ exceeds 0.0074, self-rolling velocity decreases with increasing $\bar{\beta }$.

Figure 10 illustrates the effect of the rolling damping coefficient $\bar{\beta }$ on the self-rolling behavior of the LCE-based wheel with parameters set as $\bar{I_{0}}=0.4$, $\bar{C_{0}}=0.15$, $\bar{g}=2.5$, and $\bar{M_{d0}}=0.005$. As depicted in Figure 10a, the self-rolling velocity decreases as the rolling damping coefficient increases. When the other conditions remain unchanged, an increase in the rolling damping coefficient leads to a corresponding increase in the damping torque. This higher damping torque exerts greater resistance to the self-rolling motion, thereby suppressing the wheel’s motion and ultimately reducing the self-rolling velocity. When it exceeds 0.0074, the driving torque is insufficient to overcome the damping torque, and the wheel remains static, which aligns with the theoretical prediction obtained from Equation (14). Figure 10b shows the relationship between the driving torque and self-rolling velocity for different rolling damping coefficients (0.001, 0.002, and 0.004). As the coefficient increases, the driving torque decreases because higher self-rolling velocities reduce the time the LCE rods spend in the illuminated region, limiting thermal energy absorption. The red dashed lines in Figure 10b represent the total damping torque for the different coefficients, with values of 0.0075, 0.01, and 0.015. The intersections of the driving torque and damping torque curves indicate the equilibrium points for stable self-rolling. In practical applications, increasing the damping coefficient reduces self-rolling velocity and can even hinder motion stability. Therefore, optimizing the rolling damping coefficient is crucial for achieving stable self-rolling motion, especially in systems requiring precise control over motion stability and energy efficiency.

## 5. Potential Applications

**Figure 11:**
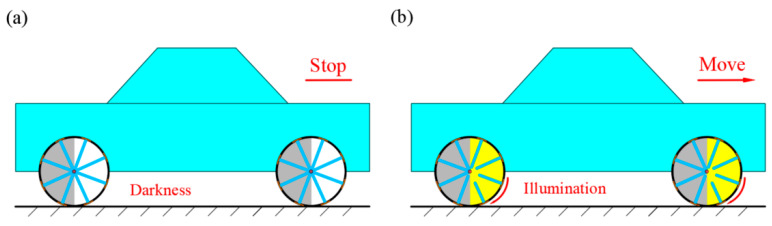
Vehicle driven by LCE-based wheels. (**a**) Dark state. (**b**) Illuminated state. Under constant illumination, vehicle of LCE-based wheels moves forward steadily.

In this section, we explore the possibility of an autonomous vehicle driven by LCE-based wheels and investigate its working principle under different illuminated conditions. As shown in Figure 11, this system demonstrates the unique behavior of the LCE-based wheels in both illuminated and dark states. In Figure 11a, the vehicle remains stationary in the absence of illumination because the LCE wheels are not activated by illumination and fail to generate a driving torque, causing the vehicle to remain immobile. In contrast, as shown in Figure 11b, when the LCE rods are exposed to illumination, the photothermal effect causes the LCE rods to contract, generating a driving torque that overcomes the damping torque in the system. This enables the vehicle to move forward steadily. This process highlights the photothermal response of the LCE material, allowing it to self-drive under illumination, thereby driving the movement of the vehicle. The potential of this technology extends beyond just driving the vehicle; it also provides a new perspective for autonomous driving systems. By utilizing an external illumination source to activate the driving capability of the LCE material, this light-fueled self-rolling mechanism offers an innovative driving approach for future automated transportation systems. It has the potential to play an important role in energy efficiency, high performance, and automation. It offers a promising direction for future transportation solutions, especially for automated systems in complex environments.

## 6. Conclusions

Despite significant advancements in LCE-based self-excited systems, traditional oscillatory systems often face challenges in terms of structural stability and increased friction, limiting their practical applications. In this study, we propose a light-fueled stable self-rolling LCE-based wheel that addresses the limitations of traditional oscillatory systems by offering improved stability and reduced friction. Through a photothermal response model, we identify the driving torque mechanism and the key parameters—heat flux, contraction coefficient, and gravitational acceleration—that influence performance. Our findings show that the wheel achieves stable self-rolling with minimal friction, maintaining a nearly constant shape that suppresses oscillations and enhances stability. This simple, low-damping system demonstrates promising potential for applications in autonomous drive systems, micro-transportation devices, and photothermal energy conversion technologies. Future work will focus on refining the contraction direction of LCE rods to optimize performance and prevent irregular deformations. However, experimental validation faces challenges, such as the need for precise control and measurement of the contraction direction under varying light and thermal conditions. The interactions between heat flux, contraction coefficients, and external forces may lead to non-uniform deformations that are difficult to measure at small scales. Despite these difficulties, we believe future experiments will offer valuable insights into optimizing LCE-based systems and advancing stable, energy-efficient self-excited technologies.

## Figures and Tables

**Table 1:** Material properties and geometric parameters.

Parameter	Definition	Value	Unit
$C_{0}$	Contraction coefficient	0~0.005	1/K
R	Initial length of LCE rods	5	cm
$M_{d0}$	Initial damping torque	0~0.001	N⋅m
$\tau_{0}$	Heat exchange time between LCE rods and environment	0.001~0.1	s
β	Rolling damping coefficient	0~0.01	cm
$I_{0}$	Heat flux	0~2 × 10^3^	W
K	Thermal conductivity coefficient	0~8	W/K
$\rho_{c}$	Specific heat capacity	0.1	J/K
$T_{e}$	Temperature of environment	300	K
g	Gravitational acceleration	0~50	${m/s}^{2}$
m	Mass	0~0.01	kg
α	Transition coefficient	10~500	/

**Table 2:** Dimensionless parameters.

Parameter	$\bar{I_{0}}$	$\bar{C_{0}}$	$\bar{g}$	$\bar{M_{d0}}$	$\bar{\beta }$
Value	0~0.8	0~1.5	0~10	0~0.004	0~0.002

## Data Availability

The original contributions presented in this study are included in the article. Further inquiries can be directed to the corresponding author.
